# Transurethral resection of the prostate in 85+ patients: a retrospective, multicentre study

**DOI:** 10.1007/s00345-022-04179-w

**Published:** 2022-10-14

**Authors:** Michael Lotterstätter, Stephan Seklehner, Florian Wimpissinger, Jozsef Gombos, Jasmin Bektic, Philipp Stolzlechner, Sarah Laimer, Thomas R. W. Herrmann, Stephan Madersbacher, Lukas Lusuardi, Manuela Sieberer, Christian Ramesmayer

**Affiliations:** 1 Department of Urology, Klinik Favoriten, Vienna, Austria; 2 Department of Urology, Landesklinikum Baden-Mödling, Baden, Austria; 3 Department of Urology and Andrology, Paracelsus Medical University, Müllner Hauptstraße 48, 5020 Salzburg, Austria; 4 Department of Urology, Landesklinikum Mistelbach, Mistelbach, Austria; 5 Department of Urology, Rudolfstiftung Hospital, Vienna, Austria; 6 Department of Urology, Landesklinikum Wiener Neustadt, Wiener Neustadt, Austria; 7 Department of Urology, Medical University Innsbruck, Innsbruck, Austria; 8 Department of Urology, Tauernklinikum Zell am See, Zell am See, Austria; 9 Department of Urology, Spital Thurgau AG, Kantonspital Frauenfeld, Frauenfeld, Switzerland; 10 Urology Stellenbosch University, Western Cape, South Africa; 11 Hannover Medical Scholl MHH, Carl Neuberg Str. 1 30625, Hannover, Germany

**Keywords:** TUR-P, Old-old, BPH, Endourology, Urinary retention

## Abstract

**Purpose:**

To determine the safety and efficacy of transurethral resection of the prostate (TUR-P) in patients 85 years or older.

**Methods:**

In this retrospective, multicentre study, patients equal or older than 85 years at the time of surgery (2015–2020) were included. Several pre-, peri- and postoperative parameters were collected. The main outcome criterion was spontaneous voiding with a post-void residual (PVR) volume < 100 ml at dismission and at 12 months after surgery.

**Results:**

One hundred sixty-eight patients (median age: 87 years, interquartile range [IQR]: 86–89) were recruited. The patients took on average 5.2 permanent medications (3–8), 107 (64%) were anticoagulated preoperatively and neurological co-morbidities were present in 29 (17%). The indication for surgery was recurrent urinary retention in 66.3% (*n* = 110) with a mean retention volume of 849 ml. The mean PVR volume of the remaining 35% was 146 ml. Surgery was successfully completed in all patients. A perioperative surgical revision had to be performed in 3% and 13 patients (7.7%) required blood transfusion. After catheter removal, 85% of patients were able to void spontaneously with a PVR < 100 ml, and 14.3% were dismissed with a catheter. Twelve months data were available for 93 patients (55%). Of this cohort, 78 (83.9%) were able to void spontaneously with a PVR < 100 ml, 12 (12.9%) were on permanent catheterization. One patient (0.6%) died perioperatively. The only significant factor associated with an unsuccessful outcome was the number of permanent medications (6.8 vs. 5.0, *p* = 0.005).

**Conclusion:**

This retrospective multicentre study documents the safety and efficacy of TURP (monopolar and bipolar) in the old-old cohort.

## Introduction

The ever-increasing elderly population is one of the major challenges in medicine and urology in particular. Austria (total population: 9.0 Mio) currently has 414.000 inhabitants equal to or older than 80 years. This number will increase to 639.000 in 2030 and to 1.1 Mio in 2060, indicating a rise of 166% [1]. These demographic changes will substantially enhance also the number of elderly patients requiring surgical intervention for benign prostatic obstruction (BPO) in upcoming decades as lower urinary tract dysfunction (LUTD) has a strong age relation [1, 2].

The indication for disobstructive surgery in elderly patients has to balance life expectancy, functional outcome and surgical risk [2]. Approximately, 12% of patients older than 75 years of age die within 12 months after the first episode of urinary retention and this rate increases up to 30% in nursing home patients who underwent TURP [2–4]. In parallel, the functional outcome decreases with advancing age with age-related detrusor underactivity being a relevant co-factor [5–7]. Finally, morbidity after TURP is increased by age, polymedication, and preoperative urinary retention.

TURP remains the by far most frequent invasive treatment for benign prostatic obstruction [8]. According to an Austrian population-based study, 12% of patients who underwent TURP in the years 2002–2006 are older than 80 years [9]. Despite this fact, the data on invasive BPO treatment in geriatric patients are rather scant and we are not aware of a single study that has specifically assessed the safety and outcome of TURP in the old-old age group, i.e. 85 years or older.

We therefore aimed to analyse this issue in a multicentre, retrospective study. We included only men aged 85 years or older at the time of surgery. The multicentre design was chosen to enhance sample size and generalizability.

## Patients and methods

### Study design

In this retrospective, multicentre study, all patients equal or older than 85 years at the time of TURP performed between 2015 and 2020 were included. There were no exclusion criteria with the exception of previous TURP and the indication of locally advanced prostate cancer. The following baseline parameters were recorded: age, indwelling catheter preoperatively, retention volume, post-void residual volume (PVR), Charlson Comorbitidy Index, medication and anticoagulation. We also collected various intra- and postoperative parameters as the modality of resection (bipolar/monopolar), histology, resection volume, operation time, discharge with/without indwelling catheter, hospitalisation days, and complications according to Clavien–Dindo. Incontinence was assessed by non-standardized questions during follow-up according to the standards of the participating centres. Follow-up data were obtained 12 months postoperatively. The main outcome criterion was spontaneous voiding with a post-void residual volume of less than 100 ml at dismission and at 12 months after surgery.

### Statistical analysis

All data of continuous variables were checked for normal distribution.

In the case of normal distribution, the independent two-sample *t* test was used for the specific subgroup comparison of two subgroups. For variables without normally distributed data, the exact Mann–Whitney *U* test was applied. Dichotomous variables were compared by the Fisher’s exact test.

For comparisons of three subgroups for continuous variables, a non-parametric analysis of variance (Kruskal–Wallis test, followed by Nemenyi’s multiple comparisons) was used. Categorical variables were compared by the Chi-square test (exact or with Monte Carlo simulation). Two-sided 95% confidence intervals (CI) were calculated according to the nature of the data (non-parametric or according to Clopper–Pearson).

The type I error was not adjusted for multiple testing. Therefore, the results of inferential statistics are descriptive only. Statistical analyses were performed using the open-source R statistical software package, version 4.0.5 (The R Foundation for Statistical Computing, Vienna, Austria).

## Results

### Patient characteristics

A total of 168 patients were included in this retrospective study (Table 1). The median patient age was 87 years, and the oldest patient was 98 years. Patients took on average 5.2 permanent medications (interquartile range IQR: 0–16), 64% were on anticoagulation, diabetes mellitus was present in 19.6% and neurologic co-morbidities in 17.2%. The patients had a median score in the Charlson Comorbidity Index of 5.5 points.

**Table 1:** Baseline and demographic data

	Total	1	2	3	4	5	6	7
*N*	168	29	9	10	34	31	18	37
Age, median (years)	87 (86–89)	87 (86–89)	87 (86–89)	86 (85–86)	87 (86–89)	87 (86–88)	88 (86–98)	87 (86–89)
Comorbidities								
Medication (count), median	5 (3–8)	5 (2–7)	4 (2–6)	6 (4–9)	5 (3–8)	4 (2–6)	5 (2–7)	5 (3–9)
Anticoagulation (Total)	107 (63.7%)	20 (69%)	6 (66.7%)	4 (40%)	22 (64.7%)	20 (64.5%)	8 (44.4%)	27 (73%)
LMWH/coumarin	16 (15%)	4 (20%)	0	0	5 (22.7)	1 (5%)	0	6 (22.2%)
NOAC	23 (21.5%)	1 (5%)	1 (16.7%)	1 (25%)	5 (22.7%)	9 (45%)	2 (25%)	4 (14.8%)
PAI	68 (63.6%)	15 (75%)	5 (83.3)	3 (75%)	12 (54.5%)	10 (50%)	6 (75%)	17 (63%)
Charlson Comorbidity Index	5.5 (5–9)	5.5 (5–8)	4.8 (5–8)	5.7 (5.5–9)	5.3 (5–7)	5.5 (5–8)	5.5 (5–8)	5.5 (5–9)
Perioperative								
Indwelling catheter preoperatively	110 (66.3%)	21 (75%)	4 (50%)	6 (60%)	23 (67.6%)	22 (71%)	13 (72.2%)	21 (56.8%)
Bipolar resection	125 (74.4%)	28 (96.6%)	9 (100%)	10 (100%)	2 (5.9%)	31 (100%)	8 (44.4%)	37 (100%)
OP time (min), median	55 (36–75)	51 (34–69)	44 (35–50)	72 (57–93)	55 (41–73)	60 (50–80)	35 (20–40)	60 (40–90)
Resected volume, median (g)	20.5 (10–35.5)	20.4 (12–31.5)	13 (11–16)	25 (9–40)	30 (23–56)	17 (7–34)		25 (15–45)
Blood transfusion (Clavien Dindo 2)	13 (7.8%)	2 (6.9%)	0	0	7 (20.6%)	2 (6.5%)	0	2 (5.4%)
Major complications (Clavien-Dindo ≥ 3b)	5 (3%)	0	0	1 (12.5%)	0	3 (9.7%)	1 (5.6%)	0
Hospitalisation (*d*), median	6 (5–8)	8 (5–10)	5 (4–6)	7 (6–8)	7 (7–7)	5 (5–7)	6 (5–7)	5 (5–6)

Headline 1–7 representing the different centers

*OP* operating; Data in parentheses are interquartile ranges; *LMWH* low molecular weight heparin, *NOAC* new oral anticoagulants; *PAI* platelet aggregation inhibitor

The indication for surgery was recurrent urinary retention requiring permanent catheterization in 65%. Prior to surgery, only 4.2% of patients had undergone urodynamic testing.

Table 1 presents the baseline data for each of the seven participating institutions. These data—in general—demonstrate a quite homogeneous patient population across the seven institutions, e.g. regarding age, co-morbidities and indication for surgery.

### Perioperative data

In 74.4% of cases, resection was performed with the bipolar technique, and 5/7 institutions almost exclusively used the bipolar technique. The median resection volume was 20.5 g (interquartile range [IQR]: 10–35), and median operation time 55 min (36–75). All patients underwent operation under general anaesthesia. Revision surgery was necessary in 5 patients (3%), and 13 patients (7.7%) required at least one blood transfusion perioperatively. In Table 1, relevant postoperative complications according to Clavien–Dindo are listed and include primarily surgical re-intervention and blood transfusion. All patients underwent the operation under general anaesthesia. There was no association between the type of anticoagulation and the risk of surgical revision.

Pathohistological review revealed prostate cancer in 35.1% of cases.

We observed a significant difference for blood transfusion rate depending on the resection technique: monopolar: 16%, bipolar 5% (*p* = 0.041). Operative revision was similar between mono- and bipolar (2.3 vs. 3.3%) techniques.

### Outcome

86% of patients were able to urinate spontaneously and with a post-void residual volume (PVR) of less than 100 ml and hence discharged without indwelling catheter. Patients were discharged after a median of 6 days (5-8). One patient died perioperatively (0.6%). Complete data with breakdown according to the seven departments are presented in Table 2. Again, the outcome data were quite homogeneous between the seven centres.

**Table 2:** Outcome

	Total	1	2	3	4	5	6	7
*N*	168	29	9	10	34	31	18	37
Age, median (years)	87 (86–89)	87 (86–89)	87 (86–89)	86 (85–86)	87 (86–89)	87 (86–88)	88 (86–98)	87 (86–89)
Discharge without catheter	144 (85.7%)	24 (82.8%)	8 (88.9%)	9 (90%)	29 (85.3%)	23 (74.2%)	17 (94.4%)	34 (91.9%)
Discharge with catheter or PVR > 100 ml	27 (17.5%)	6 (23.1%)	1 (11.1%)	1 (11.1%)	6 (17.6%)	8 (25.8%)	1 (12.5%)	4 (10.8%)
12 months post-OP: catheter free	78 (85.7%)	12 (93.3%)	5 (71.4%)	3 (100%)	26 (100%)	21 (75%)	5 (71.4%)	6 (85.7%)
12 months post-OP incontinence	5 (6%)	1 (14.3%)	0	3 (12%)	3 (12%)	0	0	0
12 months post-OP Re-Intervention	3 (3.2%)	1 (7.1%)	1 (12.5%)	0	0	0	0	1 (14.3%)

Headline 1–7 representing the different centers

*PVR* post void residual volume. Data in parantheses are interquartile ranges

Follow-up data after 12 months were available in 55% of patients. Of these 93 patients, 85.7% were able to void spontaneously with a PVR of less than 100 ml. Mean PVR was 51.3 ml. Urinary incontinence was recorded in 5.4% of the patients. A surgical re-Intervention had to be performed in three patients (3.2%).

### Correlates for successful voiding

Table 3 compares various baseline/perioperative parameters to the clinical outcome (discharge with/without a catheter). Despite some minor differences, the only parameter that reached statistical significance was the number of permanent medications, which was higher in the failure cohort (6.8 vs. 5.0, *p* = 0.005). Co-morbidities rated according to Charlson Comorbidity Index, however, did not reach significance, yet was higher in the failure cohort.

**Table 3:** Correlates for a successful voiding at dismission

	No indwelling catheter	Indwelling catheter	*p* value
Age (years)	87 (86–89)	87 (86–89)	0,22
Medication (count)	4 (2–8)	6 (4–9)	**0.005**
Anticoagulation (Total)	61.8%	75%	0.26
Charlson Comorbidity Index	5 (5–6)	5.5 (5–6)	0.7
Resection volume (g)	22 (10.5–34.5)	20 (6.5–36.2)	0.47
Neurologic comorbidities	35.3%	33.3%	> 0.99
Indwelling catheter preoperatively	64.1%	79.2%	0.17
Bipolar resection	73.6%	79.2%	0.80
OP time (min)	57.5 (40–80)	51 (31–72)	0.47
Hospitalisation (*d*)	6 (5–7)	7 (5–10)	0.8
Major complications Clavien-Dindo ≥ 3b	2.8%	4.2%	0.4

Data in parentheses are interquartile ranges

## Discussion

In summary, this study demonstrates acceptable safety and efficacy of mono/bipolar TURP in the old-old cohort. At 12 months, almost 85% of patients were able to void spontaneously with a post-void residual volume of less than 100 ml, perioperative mortality was less than 1% and morbidity acceptable. In our study, we set a threshold of 100 ml post-void residual volume, which lies below the threshold in Detrusor underactivity according to the definition of the International Continence Society [10]. Strengths of the study are the fact that it is the first that has exclusively studied men belonging to the 85+ cohort and its multicentre design. Major limitations are the retrospective design, the lack of standardized assessment of LUTS by, e.g. the International Prostate Symptom Score, the lack of geriatric scores and—probably the most relevant—the fact that 12 months follow-up data were available for only 55% of patients. One might speculate that this incomplete follow-up is partly due to the high patient age leading to no willingness or capability to perform follow-up visits. Furthermore, the rate of spinal anaesthesia and the prostate volume were not recorded.

A number of studies have documented the safety and efficacy of desobstructive surgery also in the elderly, although complication- and success rates are less favourable in this cohort [5–7]. Studies focussing on TUR-P in old men (75–80 years) reported successful catheter withdrawal in the range of 65–89%, mirroring results of the present study (86%) [5, 6]. Brierly et al. reported on 93 patients older than 80yrs (mean age: 84 years) operated 1993–1997. In their study, 31% of patients underwent TURP for symptoms and 68% for urinary retention [5]. The early and late complication rates were 41% and 22%, respectively. Of all patients with retention, 80% were able to void with small residual volumes by 6 weeks after operation [5]. Data regarding indication for TUR-P and outcome measures are quite similar to the present study, whereas morbidity was surprisingly lower in the current series. A few studies have also reported promising data on the outcome of elderly men undergoing laser prostatectomy [11, 12]. In our study there was no statistical significance regarding the bleeding risk in the different anticoagulants. Similar data were published by Deuker et al., who analysed patients under anticoagulation therapy treated with holmium laser enucleation of the prostate [13]. No higher complication rates were reported.

Optimistic results of desobstructive surgery in elderly men, both from previously published trials and the present study, are contrasted by a large study on nursing home patients. Suskind et al. studied 2.869 nursing home patients who underwent desobstructive surgery [4]. The outcome of patients with preoperative catheterization (*n* = 1.178; mean age: 80.6 years; Charlson Comorbidity Scale ≥ 2: 45.5%) at 12 months was as follows: 30% have died and of those alive, 95% remained on permanent catheterization [4]. These results show that virtually all patients were on catheterization one year after surgery indicating that TURP failed in almost all patients [4]. Although this study contains no information on the indication for re-catheterisation, it emphasises the need for a thorough preoperative work-up in geriatric patients, yet contrasts the majority on surgical series in this age cohort presented above [4].

The *eau*-guidelines on male LUTS recommend urodynamic evaluation in elderly patients (e.g. 80+) prior to prostatectomy because of the well-known age related urodynamic changes towards less obstruction and higher rates of detrusor dysfunction [14]. Despite this (yet weak) recommendation, less than 5% of men in this contemporary series underwent preoperative urodynamics. In 4 of the 7 institutions involved, not a single urodynamic study has been performed in their patients before TUR-P. Non-invasive urodynamic examinations, such as uroflowmetry, were rarely performed in the catheter-free cohort. After all, the role of urodynamics prior to surgery remains controversial [15, 16]. However, in opposite to the EAU recommendations on male LUTS, the UPSTREAM-study could not show a change of progress to surgery with prior urodynamic assessment [16].

In this multicentre series a substantial difference in the transfusion rate was observed between mono- and bipolar TUR-P. Although these data might be biased by the fact that only 2 participating centres used monopolar TUR-P, these data are in line with the literature although the *eau-*guidelines state the trial quality is limited on this topic [14, 17, 18].

Recent studies on desobstructive surgery in elderly patients suggest that (i) the outcome is strongly influenced by “geriatric factors” and (ii) that a preoperative geriatric assessment might help in the decision-making process [1, 19]. Knoblauch et al. have shown that predictors for the outcome of elderly patients following laser ablation are “geriatric factors” such as frailty, co-morbidities and co-medication rather than the conventional urological parameters [11]. In our series, the number of co-medication was the only significant risk factor associated with treatment failure (6.8 vs 5.0, *p* = 0.005). Interestingly, holmium laser enucleation is not associated with prolongued catherization in patients over 80+ . These data were recently published by Gild et al. [20]. This might represent a point for complete desobstruction in these frail cohort in contrast to channel-TUR-P. Two recent prospective studies suggest that elderly patients with good performance status profit from surgery in a comparable way as younger patients do [1, 21]. In contrast elderly frail patients have a worse outcome and might therefore profit form a more in-depth evaluation [1]. Elderly, frail patients should be offered minimally invasive treatment, such as water vaporisation or prostate artery embolization, that can be performed under local anaesthesia [21, 22]. Eredics et al. recently reported on a multicentre study of 136 patients with recurrent urinary retention who underwent water vapor therapy in an ambulatory setting with periprostatic block and optional sedation [23]. After 3 and 12 months, 93.5 and 91% of patients remained catheter independent, thus confirming results of a previous smaller sized series [23, 24]. These functional data are quite similar to those of the above cited studies following conventional desobstructive surgery performed under general anaesthesia suggesting that some forms of minimal invasive procedures seem to be suitable in this particular cohort.

## Conclusions

For the first time, acceptable safety and efficacy of mono/bipolar TURP for BPO could be demonstrated in the old-old cohort. At 12 months following TUR-P, 85% of patients were able to void spontaneously with a post-void residual volume of less than 100 ml, perioperative mortality was less than 1% and morbidity low.
